# Inhibition of Bromodomain and Extra Terminal (BET) Domain Activity Modulates the IL-23R/IL-17 Axis and Suppresses Acute Graft-*Versus*-Host Disease

**DOI:** 10.3389/fonc.2021.760789

**Published:** 2021-10-15

**Authors:** Katiri J. Snyder, Hannah K. Choe, Yandi Gao, Natalie E. Sell, Kara M. Braunreiter, Nina C. Zitzer, Lotus Neidemire-Colley, Sonu Kalyan, Adrienne M. Dorrance, Andrea Keller, Maria M. Mihaylova, Satishkumar Singh, Lalit Sehgal, Gideon Bollag, Yan Ma, Ben Powell, Steven M. Devine, Parvathi Ranganathan

**Affiliations:** ^1^ Division of Hematology, Department of Internal Medicine, The Ohio State University, Columbus, OH, United States; ^2^ Department of Biological Chemistry and Pharmacology, Comprehensive Cancer Center, The Ohio State University Columbus, Columbus, OH, United States; ^3^ Plexxikon Inc, South San Francisco, CA, United States

**Keywords:** GVHD, IL-23R, T cells, GVHD biology, BET (Bromodomain and extra-terminal) inhibitor

## Abstract

Acute graft-*versus*-host disease (GVHD) is the leading cause of non-relapse mortality following allogeneic hematopoietic cell transplantation. The majority of patients non-responsive to front line treatment with steroids have an estimated overall 2-year survival rate of only 10%. Bromodomain and extra-terminal domain (BET) proteins influence inflammatory gene transcription, and therefore represent a potential target to mitigate inflammation central to acute GVHD pathogenesis. Using potent and selective BET inhibitors Plexxikon-51107 and -2853 (PLX51107 and PLX2853), we show that BET inhibition significantly improves survival and reduces disease progression in murine models of acute GVHD without sacrificing the beneficial graft-*versus*-leukemia response. BET inhibition reduces T cell alloreactive proliferation, decreases inflammatory cytokine production, and impairs dendritic cell maturation both *in vitro* and *in vivo*. RNA sequencing studies in human T cells revealed that BET inhibition impacts inflammatory IL-17 and IL-12 gene expression signatures, and Chromatin Immunoprecipitation (ChIP)-sequencing revealed that BRD4 binds directly to the IL-23R gene locus. BET inhibition results in decreased IL-23R expression and function as demonstrated by decreased phosphorylation of STAT3 in response to IL-23 stimulation in human T cells *in vitro* as well as in mouse donor T cells *in vivo*. Furthermore, PLX2853 significantly reduced IL-23R+ and pathogenic CD4+ IFNγ+ IL-17+ double positive T cell infiltration in gastrointestinal tissues in an acute GVHD murine model. Our findings identify a role for BET proteins in regulating the IL-23R/STAT3/IL-17 pathway. Based on our preclinical data presented here, PLX51107 will enter clinical trial for refractory acute GVHD in a Phase 1 safety, biological efficacy trial.

## Introduction

Acute graft-*versus*-host disease (GVHD) is a T cell mediated disorder commonly associated with allogeneic hematopoietic cell transplantation (HCT) leading to transplant-related mortality. Acute GVHD occurs when donor T cells recognize host minor and major histocompatibility complex (MHC) antigens as non-self and mount an inflammatory immune response involving multiple steps: immune response priming by conditioning regimen-induced tissue damage, donor T cell activation, T cell expansion and differentiation, T cell migration to target tissues, and destruction of target tissues by inflammatory cytokines and direct cytotoxicity (1–6). Tissue damage associated with pre-transplant conditioning chemotherapy or radiation induces the secretion of a multitude of cytokines including tumor necrosis factor (TNF), interleukin 1 (IL-1), IL-12, IL-23 and IL-6 which leads to the activation of host antigen presenting cells (APC). APC subsequently activate donor T cells from the graft, further propagating the release of inflammatory cytokines such as IL-17, IFN-γ, and TNF that ultimately results in target tissue destruction (4). Th17-associated cytokines such as IL-17 and IL-23 are known to be increased in GVHD, highlighting the importance of this T cell subset in the pathogenesis of the disorder (7–11). Additionally, loss of host gastrointestinal (GI) tissue integrity is of particular importance as GI damage permits the translocation of microbial products which trigger further host APC activation, resulting in a vicious cycle of rampant T cell activation and subsequent tissue destruction (2, 12–15).

Bromodomain and extra-terminal domain (BET) proteins, namely BRD2, BRD3, BRD4, and BRDT, are a conserved family of epigenetic regulators that, *via* their two bromodomains BD1 and BD2, regulate gene transcription by binding to acetylated lysine residues on histones. BET proteins have been shown to bind specifically to genes associated with super-enhancers enriched with acetyl-histone H3K27 (H3K27Ac) (16, 17). Aberrant expression of BET proteins has been shown to contribute to carcinogenesis by mediating hyperacetylation on regions of chromatin that contain proliferative genes (17). BRD4 in particular serves as an epigenetic reader responsible for linking active chromatin marks to transcriptional elongation by the activation of RNA polymerase II (17).

Pharmacological inhibition of the BET family of proteins is a promising targeted treatment for cancers including acute leukemia (18, 19), myelodysplastic syndrome (NCT04022785), progressive lymphoma (NCT01949883, NCT03936465, NCT03068351, NCT04022785, NCT04116359), NUT cancer (NCT04116359), prostate cancer, CLL (20), and non-small cell lung cancer (21, 22). BET inhibition also potently suppresses the Th17-mediated inflammatory response, achieved by impairment of T cell differentiation and proliferation (23–25). Inhibition of BET proteins has been shown to modulate the inflammatory response of both dendritic cells and T cells potentially due to the disruption of the interaction between BRD4 and acetyl-310 RelA of NF-κB (26). BET inhibition in an allogeneic bone marrow transplantation mouse model has been shown to reduce the severity of GVHD and improve overall survival (26, 27); however, clinical translation of these agents is severely limited by lack of single agent efficacy in conjunction with broad target specificity and/or short half-life (26–30).

Therefore, we employed the use of the structurally distinct BET inhibitors, Plexxikon-51107 and -2853 (PLX51107 and PLX2853), that were modified from in-house screening hits with weak affinity and optimized to generate compounds with high affinity and selectivity and are structurally distinct from other identified and reported BET family inhibitors. Specifically, PLX51107 and PLX2853 exhibit unique binding by a novel 7-azaindole scaffold as opposed to the more common benzodiazepine scaffold of previous BET inhibitors (20). These inhibitors bind to acetylated lysine motifs in the bromodomains of BRD4 preventing the binding of BRD4 to its target acetylated lysines on histones and other proteins. PLX51107 and PLX2853 exhibited a unique binding mode, differentiated pharmacokinetic (PK) profile, and improved tolerability. In a substrate binding assay using engineered BRD2 and BRD4 proteins that contain both bromodomains, PLX2853 was shown to be 7-9-fold more potent than PLX51107 (**Table 1**). The binding of either PLX51107 or PLX2853 to BRD4 results in inhibition of RelA binding and the downstream transcriptional activation of inflammatory response genes (20, 31, 32).

**Table 1:** IC_50_ values for PLX51107 and PLX2853 against BET proteins.

BET Proteins	PLX51107		PLX2853	
	IC_50_ (μM)^a^	CI_95_ (μM)^b^	IC_50_ (μM)^a^	CI_95_ (μM)^b^
BRD2-BD12	0.052	0.046-0.059	0.0073	0.0035-0.015
BRD4-BD12	0.023	0.021-0.026	0.0043	0.0026-0.007

a Geometric mean of the IC_50_.

b 95% Confidence interval for geometric mean.

Both PLX51107 and PLX2853 compounds possess promising pharmacokinetic profiles in rodents and humans: high peak plasma compound concentrations and short terminal half-life. Here, we examine the therapeutic potential of this novel class of BET inhibitor for treating acute GVHD, anticipating efficient translation into clinical studies. Overall, our results show that BET inhibition by PLX51107 and PLX2853 represents a promising treatment strategy for acute GVHD.

## Materials And Methods

### Mice

C57BL/6 (B6, H2^b^), B6.SJL-Ptprc^a^ Pepc/BoyJ (CD45.1 B6), B6D2F1 (F1, H2^b/d^), C3.SW-*H2^b^*/SnJ (C3.SW, H2^b^), and BALB/c (H2^d^) mice were purchased from Jackson ImmunoResearch Laboratories (Bar Harbor, ME). For transplant experiments, recipient mice were between 12 and 16 weeks of age. For all other experiments, mice were between 8-10 weeks of age. All animal studies were conducted in accordance with the rules and regulations of the Institutional Animal Care and Use Committee at OSU.

### Acute GVHD Murine Models

Mice were transplanted under standard protocols approved by the University Committee on Use and Care of Laboratory Animals at OSU. Only age- and sex-matched mice were used for transplant experiments. Three separate models were used for *in-vivo* acute GVHD studies. In the first model, B6D2F1 mice were irradiated with 1200 cGy administered in 2 fractions (to minimize toxicity) one day before transplant. T cell depleted bone marrow (TCD-BM) cells [10x10 [6]] plus [15x10 [6]] total splenocytes from CD45.1 B6 donors were administered on the day of transplant. T cell depletion from BM cells was carried out by CD90.2 magnetic bead separation (Miltenyi Biotec). In the second model of minor histocompatibility antigen (miHA) mismatched experiments, B6 recipient mice were irradiated with 1000 cGy in a single dose on the day of transplant. C3.SW donor CD8 T cells [1x10 [6]] and TCD-BM cells [10x10 [6]] were administered. In the third model BALB/c recipients underwent, recipients underwent 700 cGy irradiation fractionated into two doses and received [0.7x10 [6]] B6 T cells plus [10x10 [6]] TCD-BM cells on the following day. All cell infusions were administered *via* tail vein injection. Recipients of allogeneic splenocytes or T cells were treated with vehicle, BET inhibitor PLX51107 (10 mg/kg) or PLX2853 (3mg/kg), administered by oral gavage three times weekly starting day +1 or day +7 post-transplant until the end of the study. For the B6 into BALB/c model alone, treatment was initiated at day +10 due to the inherent fragility of this model leading to early deaths in the first week post-transplant; only those mice surviving past day 10 were used for the study.

### PLX51107 and PLX2853

BET inhibitors PLX51107 and PLX2853 were developed by Plexxikon. The compounds were dissolved in N-Methyl-2-pyrrolidone (NMP) and administered into a final formulation of 10% NMP plus diluent (40% PEG400, 5% TPGS, 5% Poloxamer 407 and 50% Water). Mice received 10 mg/kg PLX51107 or 3 mg/kg PLX2853 three times per week *via* oral gavage beginning day +1 or day +7 post-transplant until the end of the study.

### Clinical and Histologic Assessment of Acute GVHD

Recipient mice were weighed 2-4 times a week and monitored daily for clinical signs of acute GVHD and survival. A scoring method adapted and modified from Cooke et al. (33) was used to assess clinical changes associated with aGVHD. Briefly, this scoring system incorporates 5 clinical parameters: weight loss, posture (hunching), activity, fur texture, and skin integrity. Individual mice were ear tagged and graded (in a scale from 0 to 8) twice a week. Mice who reached an acute GVHD score of more than or equal to 7 were very sick and were euthanized and their tissues harvested. GVHD was also assessed by detailed histopathology analysis of H&E stained liver and gut tissues using a previously reported scoring system with a range of 0 (absence of signs of GVHD) to 4 (maximal GVHD damage) (34). A separate cohort of mice were euthanized around day 25 (± 3 days) post-transplant and used for histopathological assessment of target tissues.

### Flow Cytometry Analysis

Around day 28 post-BMT, cohorts of mice were euthanized and splenocytes and small intestine were harvested for flow cytometric analysis. Intraepithelial lymphocytes (IELs) were isolated from lamina propria and digested into a single cell suspension using a commercial mouse Lamina Propria Tissue Dissociation Kit (Miltenyi Biotec). To select only the donor T cells, a specific gating strategy was used (**Figure S7**). A complete list of antibodies used is listed in **Table S1**. For cytokine evaluation, splenocytes and IELs were incubated for 5 hours with eBioscience Cell Stimulation Cocktail (plus protein transport inhibitors, Thermo Fisher Scientific) for T cell stimulation and protein transport inhibition. Cells were then stained with surface antibodies, permeabilized, fixed, stained with intracellular antibodies and analyzed within 24 hours. Analysis was performed with a FACS LSRFortessa flow cytometer; FACSDiva software (Becton Dickinson), and data analysis was performed using FlowJo (Tree Star).

### Immunohistochemistry

Tissues were fixed in 10% formalin overnight, paraffin embedded, and sectioned. Deparaffinized sections were subjected to antigen retrieval using Borg Decloaker RTU solution (Biocare Medical) in a pressure cooker (Instant Pot) for 20 minutes. Sections were incubated with rabbit anti-Ki-67 (D3B5) primary antibody (1:400, CST 12202S) overnight at 4°C. Secondary antibody used was biotin-conjugated donkey anti-rabbit (Jackson ImmunoResearch), followed by detection using the Vectastain Elite ABC immunoperoxidase detection kit (Vector Labs) and Dako Liquid DAB+ Substrate (Dako). Sections were counterstained with hematoxylin and mounted with Cytoseal XYL (Thermo) for visualization. Antibody dilutions were made in Common Antibody Diluent (BioGenex). Number of crypts with >10 Ki-67 positive cells were averaged across 0.5 cm of tissue.

### *In Situ* Hybridization

Single-molecule *in situ* hybridization was performed using RNAscope 2.5 HD Assay-Red (Advanced Cell Diagnostics) according to manufacturer protocol with the *Mm-Lgr5* probe. Number of cells per crypt with *Lgr5* positive staining were averaged across 50 crypts.

### GVL Experiments

B6D2F1 recipients were lethally irradiated (1200 cGy) in two doses to minimize toxicity on day -1. Firefly luciferase transduced P815 mastocytoma cells (2,000) were injected intravenously into F1 recipients on day 0 along with TCD-BM [10x10 [6]] cells. B6 donor splenocytes [15 x 10 [6]] cells were administered intravenously on day +1 to treatment groups. Treatment groups included vehicle and BET inhibitor PLX2853 3mg/kg, administered by oral gavage three times weekly starting day +2 post-transplant. TCD-BM and P815 cells (leukemia alone) served as the control group. P815-induced leukemic death was defined by the occurrence of either macroscopic tumor nodules in liver and/or spleen or hind-leg paralysis. GVHD death was defined by the absence of leukemia and the presence of clinical and histopathological signs of GVHD.

### *In Vivo* Imaging

Xenogen IVIS imaging system (Caliper Life Sciences) was used for live animal imaging. Mice were anesthetized using 1.5% isofluorane (Piramal Healthcare). XenoLight RediJect D-Luciferin Ultra Bioluminescent Substrate (150 mg/kg body weight; 30 mg/mL in PBS; Perkin Elmer) was injected intraperitoneally and IVIS imaging was performed 7-10 minutes after substrate injection. Whole body bioluminescent signal intensity was determined using IVIS Living Image software v4.3.1 (Caliper Life Sciences), and pseudocolor images overlaid on conventional photographs are shown. Data were analyzed and presented as photon counts per area.

### STAT3 Assays

PBMCs [1x10 (6) cells/mL] were cultured in RPMI-1640 containing 20% FBS and 1% PSG with CD3/CD28 DynaBeads for 48 hrs in the presence or absence of PLX51107 (250nM) or PLX2853 (10nM). On day 3, CD3/CD28 DynaBeads were removed, cells pelleted by centrifugation, resuspended and incubated in RPMI-1640 containing 1% FBS for 4-hour starvation at 37°C. Cells were then stimulated for 15 min with IL-23 (10 ng/mL, Sigma Aldrich) for STAT3 phosphorylation. For baseline level assessment, cells were left unstimulated for 15 min at 37°C. Cells were subsequently fixed with paraformaldehyde (1.5%) for 15 min and stained for the cell surface markers CD3 and CD4. After washing, cells were permeabilized with 90% ice-cold methanol for 30 min in 4°C and stained for the total and phosphorylated STAT3. Cells were analyzed on the LSRII (BD Biosciences) within 24 hours. Data analysis was performed using Flow Jo software (Tree Star).

### Degranulation Assay

CD8 T cell degranulation was measured by intracellular production of IFN-γ and CD107a in response to *ex vivo* PMA/ionomycin stimulation. Splenocytes harvested from vehicle and PLX2853 treated mice (n=6 each) were stimulated with eBioscience Cell Stimulation Cocktail (plus protein transport inhibitors, Thermo Fisher Scientific), stained for CD107a, and then incubated for 5 hours. After stimulation, the cells were stained with CD45.1, CD3 and CD8 antibodies, followed by permeabilization and staining for intracellular IFN-γ. Cells were analyzed by flow cytometry. CD45.1 donor CTL were gated on for analysis.

### mRNA-seq

Human T cells were isolated from healthy donor PBMCs (n=4 donors) by negative selection. T cells were treated with vehicle (DMSO) or PLX51107 (100 nM) for 48hrs and RNA was isolated using Trizol reagent (Invitrogen, Carlsbad, CA) and treated with DNase (Qiagen, Hilden, Germany). RNA quality was verified using the Agilent 2100 Bioanalyzer (Agilent Technologies, Santa Clara, CA) and the RNA integrity number values were greater than 7 for all samples. Sequencing libraries were generated with polyA+ RNA using the TruSeq RNA sample prep kit (Illumina, San Diego, CA). Libraries underwent paired end 50bp sequencing using the Illumina HiSeq2500 sequencer to a depth of 17 – 20 million passed filter clusters per sample. RNA-Seq data was analyzed using Basepair software (https://www.basepairtech.com/) with a pipeline that included the following steps. Reads were aligned to the transcriptome derived from UCSC genome assembly (((hg19))) using STAR (35) with default parameters. Read counts for each transcript was measured using featureCounts (36). Differentially expressed genes were determined using DESeq2 (37) and a cut-off of 0.05 on adjusted p-value (corrected for multiple hypotheses testing) was used for creating lists and heatmaps, unless otherwise stated. GSEA was performed on normalized gene expression counts, using gene permutations for calculating p-value. The data supporting the results of this article are available in the GEO repository (*accession ID: GSE183884).* (https://www.ncbi.nlm.nih.gov/geo/query/acc.cgi?acc=GSE183884).

### ChIP-Sequencing

Healthy donor (HD) T cells were stimulated with CD3/CD28 ± PLX2853 (10nM) for 24 hours. Cells were processed for chromatin immunoprecipitation (ChIP) per Active Motif kit instructions (Active Motif). ChIP sequencing (ChIP-seq) was performed using FactorPath ChIP-Seq technology by Active Motif. The data supporting the results of this article are available in the GEO repository (*accession ID: GSE183883).* (https://www.ncbi.nlm.nih.gov/geo/query/acc.cgi?acc=GSE183883).

### Chromatin Immunoprecipitation (ChIP) and Quantitative RT-PCR (qRT-PCR)

Chromatin immunoprecipitation was performed using SimpleChip enzymatic chromatin IP kit (Cell Signaling Technology) according to manufacturer’s protocol. Briefly, cells were crosslinked by incubation with 1% formaldehyde (Sigma Aldrich) at 37°C for 15 minutes. The reaction was stopped by addition of glycine (0.125 M final concentration; Sigma Aldrich). Cells were washed twice with ice-cold PBS containing 1X protease inhibitors cocktail (Cell Signaling Technology). Nuclei were isolated in 1X buffer B. The nuclei were collected and resuspended in 1X ChIP buffer then subjected to sonication using a Cole Palmer Ultrasonic homogenizer at 70% amplitude for 1 min with 1 min incubation at ice for seven cycles. The released chromatin was pelleted by centrifugation at 10,000 g for 15 minutes. Eighty to 100 μg of chromatin were pre-cleared with ChIP grade protein G magnetic beads (Cell Signaling Technology) in 1X ChIP buffer, for 3 hours at 4°C. Magnetic beads were pelleted using magnetic stand and the supernatant was incubated with ChIP grade BRD4 antibody (Active Motif) at 4°C overnight with rotation. The chromatin-antibody complexes were then bound to ChIP grade protein G magnetic beads for 2 hours at 4°C with rotation followed by pelleting of chromatin-antibody-magnetic beads complex using magnetic stand. The immune complexes were eluted with 1X ChIP elution buffer at 65°C for 30 minutes followed by reversal of cross-links at 65°C overnight. Eluted complexes were treated with RNAse A at 37°C for 2 hours and subsequently by Proteinase K at 55°C for 2 hours followed by DNA purification using DNA purification columns (Cell Signaling Technology). The purified DNA was eluted in 25 μL 1X elution buffer and 2 μL were used in qRT-PCR. Quantitative-PCR was carried out on a CFX96 Real-Time PCR System (Bio-Rad) using SYBR green PCR master mix (Applied Biosystems). The following primers were used for ChIP quantitative PCR: IL23R forward primer: TCACTGCAACCTCTGCTT, Reverse primer: GTGCCCGATGCCTGTAAT.

### Statistical Analysis

Survival data were analyzed using Kaplan-Meier and log-rank test methods. Differences between continuous variables at a single time point were analyzed using two-sided *t* tests. One-way analysis of variance (ANOVA) with Dunnet post-hoc test was used for comparisons >2 groups. For estimating statistical significance in clinical scores using multiple t-tests over time, P values were adjusted using the two-stage linear step-up procedure of Benjamini, Krieger, and Yekutieli (38). Data represent mean ± SD. All analyses were performed using GraphPad Prism 8.0. *p<0.05, **p<0.01, ***p<0.001, NS non-significant.

Additional information can be found in **Supplementary Methods**.

## Results

### BET Inhibitor PLX51107 Significantly Improves Survival in Multiple Mouse Models of Acute GVHD

**Figure 1:**
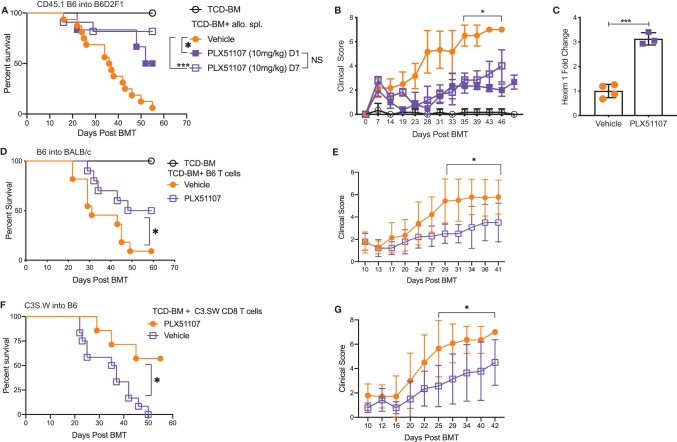
BET inhibitor PLX51107 significantly improves survival in mouse model of aGVHD. **(A)** Lethally irradiated F1 recipients received T cell depleted bone marrow cells (TCD-BM) along with CD45.1 B6 splenocytes transferred intravenously. Recipient mice were treated with vehicle (n=16) or PLX51107 (10mg/kg) by oral gavage three times weekly starting either at day day +1 (n=6) or day +7 (n=11) post-transplant. Survival curve. Log-rank test was used to compare survival. **(B)** Clinical scores. **(C)** Mice were bled after one week of dosing, six hours after last dose. Hexim1 expression in PBMCS by quantitative real-time PCR. **(D)** Lethally irradiated BALB/c recipients received TCD-BM along with T cells from B6 donors. Recipients were treated three times weekly by oral gavage with PLX51107 (n=10) or vehicle (n=11). Survival curve. **(E)** Clinical scores. **(F)** Lethally irradiated B6 recipients received TCD-BM plus CD8 T cells from C3.SW donor mice. Recipients were treated with PLX51107 (n=7) or vehicle (n=12) by oral gavage three times weekly beginning day +7 post-transplant. Survival curve. **(G)** Clinical scores. All murine transplant experiments were performed independently 2-3 times with n=3-8 mice per cohort in each experiment, data pooled from independent transplant experiments. *p < 0.05, ***p < 0.001.

Small molecule BET inhibitors PLX51107 and PLX2853 both efficiently suppressed mouse and human T cell proliferation (**Figures S1A–D**), inflammatory cytokine IFN-γ, IL-6, and TNF-α secretion (**Figures S1E–G**) as well as murine dendritic cell maturation (**Figures S2A–C**) *in vitro*. We then asked whether administration of PLX51107 to mice after allogeneic bone marrow transplantation could improve overall survival and reduce clinical severity of acute GVHD. Lethally irradiated B6D2F1 mice received allogenic splenocytes and T cell depleted bone-marrow cells (TCD-BM) from CD45.1 B6 mice. Recipients were treated with PLX51107 (10 mg/kg) or vehicle, and a cohort of B6D2F1 mice receiving CD45.1 B6 TCD-BM alone were used as controls. Mice treated with BET inhibitor PLX51107 exhibited significantly improved survival in both starting day +1 and day +7 dosing cohorts compared to mice receiving vehicle treatment (**Figure 1A**). In addition, we observed significantly reduced acute GVHD clinical scores in these mice as compared to vehicle treated recipients (**Figure 1B**). The BRD4 protein functions in opposition to hexamethylene bisacetamide inducible protein 1 (HEXIM1) by recruiting positive transcription elongation factor b (P-TEFb) to nearby promoters in order to activate transcription. HEXIM1 traps P-TEFb in an inactive complex thereby preventing this process (39). Due to the inverse relationship between these two proteins, upregulation of *Hexim1* gene transcription serves as a robust pharmacodynamic marker for BRD4 inhibition. As expected, we observed a significant upregulation of *Hexim1* gene expression in mice treated with PLX51107 indicating effective target engagement (**Figure 1C**). We also tested PLX51107 in a B6 into BALB/c model of acute GVHD. Lethally irradiated BALB/c mice received TCD-BM combined with T cells from B6 donors, followed by treatment with vehicle or PLX51107. PLX51107-treated recipients exhibited significantly improved survival as well as lower acute GVHD clinical scores (**Figures 1D, E**). We also investigated the effect of PLX51107 in a murine minor histocompatibility antigen mismatched acute GVHD model. Lethally irradiated B6 mice received C3.SW TCD-BM and CD8 T cells isolated from splenocytes and lymph nodes. Recipients of CD8 T cells were treated with vehicle or PLX51107 beginning at day +7 post-transplant. PLX51107-treated cohorts exhibited significantly improved survival and acute GVHD clinical scores compared to vehicle recipients (**Figures 1F, G**). Collectively, our data show that PLX51107 mitigates acute GVHD consistently across models with differing degrees of donor-recipient compatibility.

### Second Generation BET Inhibitor PLX2853 Improves Survival in Mouse Model of Acute GVHD

Detailed structural analyses have now determined that both bromodomains of BET proteins (BD1 and BD2) are cooperatively involved in the recognition of acetyl lysine epitopes, the interaction of inhibitors with either domain can be fine-tuned to bring about changes in both pharmacological activity and tolerability (21). Previous generations of BET inhibitors have exhibited a pan activity profile coupled with a long terminal half-life. This has resulted in general, poor clinical activity due to overt toxicity. The unique chemotype and short terminal half-life of PLX51107/PLX2853 are associated with an improved tolerability profile and a more attractive option to explore clinically.

**Figure 2:**
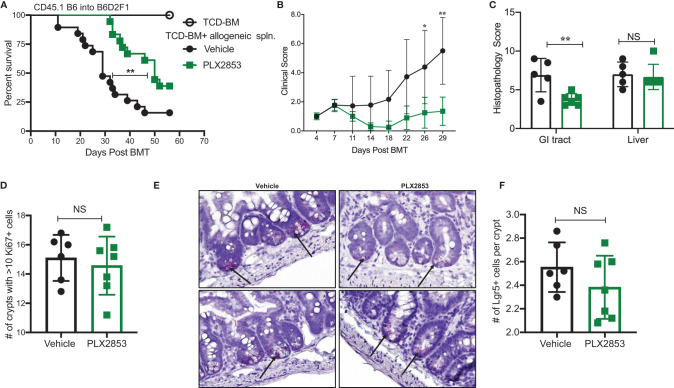
PLX2853 improves survival in mouse model of aGVHD. Lethally irradiated F1 recipients received TCD-BM only (n=10) or TCD-BM + CD45.1 B6 splenocytes. Recipients of allogeneic splenocytes (n=18-19 each) were treated with PLX2853 or vehicle by oral gavage three times weekly starting day +1 post-transplant. **(A)** Survival curve. Log-rank test. **(B)** Clinical scores. Mice were sacrificed between day 25-28 post-transplant, (n=5–6 per group) and **(C)** Histopathological score of H&E-stained liver and GI tract tissue sections, **(D)** Ki67 quantification between Vehicle and PLX2853. **(E)** Lgr5+ In-situ hybridization. **(F)** Lgr5+ analysis. Data combined from three independent transplant experiments. *p < 0.05, **p < 0.01. NS, not significant.

PLX2853 is a more potent analog of PLX51107 that exhibits low nM growth inhibition across panels of cancer cell lines spanning both leukemia and lymphoma histologies (**Table 2**). PLX2853 is currently being evaluated clinically in Phase 1 and 2 studies spanning AML/MDS, gynecologic cancer, prostate cancer and other solid tumor indications (NCT03787498, NCT03297424, NCT04493619 and NCT04556617). We tested the effects of this inhibitor in murine acute GVHD using the previously described B6 into B6D2F1 model, wherein recipients of allogeneic splenocytes were treated with PLX2853 (3 mg/kg) or vehicle by oral gavage three times per week starting on day +1 post-transplant. We observed significant prolongation of survival as well as reduced acute GVHD clinical scores and reduced liver and GI histopathology scores in mice treated with PLX2853 (**Figures 2A–C**). A previous study investigating BRD4 inhibition reported that short hairpin RNA-mediated BRD4 silencing disrupted intestinal cell proliferation as well as altered the presence of Lgr5+ intestinal stem cells (40) while a later study showed that pharmacological BET inhibition did not alter this cell population (41). Given these conflicting reports, and the established primacy of the intestinal turnover in acute GVHD pathogenesis (13, 14, 42, 43), we aimed to determine the impact of PLX2853 BET inhibition on Lgr5+ intestinal stem cells. Small intestine sections from mice euthanized around day 25 post-transplant were analyzed for Ki67+ cells by immunohistochemistry as a marker of proliferation (**Figure 2D**) and for number of Lgr5+ stem cells per crypt (**Figures 2E, F**). We observed no difference between vehicle and PLX2853 cohorts in the number of proliferating Ki67+ crypts or the number of Lgr5+ intestinal stem cells per crypt, indicating that BET inhibition does not adversely impact intestinal stem cell recovery or proliferation (**Figures 2D–F**).

**Table 2:** IC_50_ values for the growth inhibition of hematologic malignant cells by PLX51107 and PLX2853.

Cell Line	Histologic Type	PLX51107		PLX2853	
		IC_50_ (μM)^a^	CI_95_ (μM)^b^	IC_50_ (μM)^a^	CI_95_ (μM)^b^
DOHH2	Lymphoma, diffuse large B-cell	0.28	0.24-0.33	0.017	0.016, 0.018
HEL 92.1.7	Leukemia, acute erythroid	0.36	0.33, 0.39	0.042	0.034-0.054
Kasumi-1	Leukemia, acute myeloblastic	0.074	0.067-0.083	0.0043	0.0037-0.0051
MEC-1	Leukemia, chronic B cell	0.18	0.14-0.23	0.023	0.020, 0.026
MEC-2	Leukemia, chronic B cell	0.72	0.58-0.89	0.11	0.089, 0.13
MM.1S	Multiple myeloma	0.22	0.17-0.29	0.016	0.011-0.025
MUTZ-8	Leukemia, acute myeloid	1.4	1.3-1.6	0.11	0.11, 0.11
MV-4-11	Leukemia, acute myeloid	0.062	0.056-0.069	0.0041	0.0035-0.0048
NALM-6	Leukemia, acute lymphoblastic	0.14	0.14, 0.14	0.0096	0.0061-0.015
OCI-AML-2	Leukemia, acute myeloid	0.13	0.12-0.15	0.011	0.010, 0.011
OCI-AML-3	Leukemia, acute myeloid	0.071	0.068, 0.074	0.0049	0.004-0.0061
OCI-LY3	Lymphoma, diffuse large B-cell	0.4	0.36-0.46	0.026	0.017-0.041
P12-ICHIKAWA	Leukemia, precursor T-cell acute lymphoblastic	0.32	0.27-0.38	0.018	0.017, 0.020
RAMOS (RA1)	Lymphoma, Burkitt	0.64	0.56-0.73	0.037	0.036, 0.038
SET-2	Leukemia, acute megakaryoblastic	0.15	0.13-0.17	0.011	0.011, 0.012
YNH-1	Leukemia, acute myeloid	0.14	0.12-0.15	0.011	0.010,0.012

a Geometric mean of the IC_50_.

b 95% Confidence interval for geometric mean if n ≥ 3 or individual IC_50_ if n<3.

### PLX2853 Reduces T Cell and Dendritic Cell Inflammatory Response *In Vivo*

**Figure 3:**
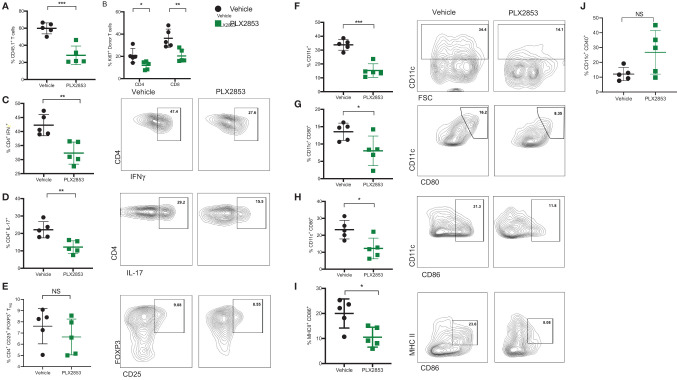
BET inhibitor PLX2853 reduces T cell and DC inflammatory responses *in vivo*. B6D2F1 recipients were lethally irradiated and received TCD-BM and splenocytes from CD45.1 B6 mice. Recipients were treated with vehicle or PLX2853 as described in methods. On days 25-28 post-transplant, cohorts of mice (n=5 each) were euthanized and splenocytes were harvested for *ex vivo* flow cytometry experiments. Percentage of **(A)** CD45.1 donor T cell in spleens, **(B)** Ki67+ donor T cells. **(C–E)** Splenocytes were stimulated with PMA/ionomycin plus protein transport inhibitors to stimulate cytokine secretion. Percentages of donor CD4+ IFNγ+, CD4+ IL-17+, and Treg populations are shown. Percentages of **(F)** CD11c+ DC, **(G)** CD80+, **(H)** CD86+, **(I)** CD86+ MHC II+ DCs and **(J)** CD40+ DCs. Data shown is representative of one of two independent transplant experiments. *p < 0.05, **p < 0.01, ***p < 0.001. NS, not significant.

We next sought to further characterize the impact of BET inhibition on T cell and dendritic cell (DC) function *in vivo*, using the B6 into B6D2F1 model as described previously. Splenocytes from mice receiving allogeneic B6 splenocytes were evaluated by flow cytometry to assess the effect of PLX2853 on T cell proliferation and inflammatory response. We observed a significant reduction in percent donor CD45.1 T cells, donor CD4 and CD8 T cells expressing proliferation marker Ki67, and donor CD4+ T cells secreting cytokines IFN-γ and IL-17 in the splenocytes of mice that were treated with PLX2853 (**Figures 3A–D**). Interestingly, we did not detect a difference in percent donor CD4+ CD25+ Foxp3+ Treg cells, suggesting that BET inhibition preserves immune tolerance (**Figure 3E** and **Figure S3**). Since our *in vitro* assays elucidated a role for BET inhibition in DC activation and maturation, we next asked if this inhibitory effect on DCs could be observed *in vivo*. We observed a significant reduction in percent total CD11c DCs in splenocytes from mice treated with PLX2853 (**Figure 3F**) accompanied by significantly reduced percentages of CD80+, CD86+, and CD86+ MHCII+ double positive DCs (**Figures 3G–I**), but not CD40 (**Figure 3J**) in splenic DCs. In all, these data bolster our *in vitro* findings and suggest that BET inhibition can have a dual effect in ameliorating acute GVHD *in vivo* by dampening both DC maturation and alloreactive T cell immune response.

### PLX2853 Retains the Beneficial Graft-*Versus*-Leukemia (GVL) Effect

**Figure 4:**
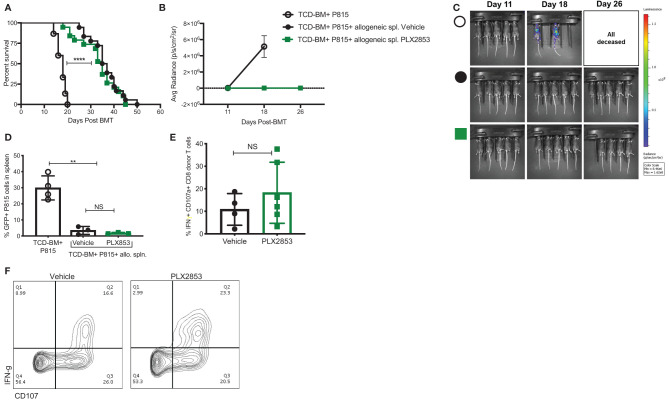
BET inhibitor PLX2853 retains GVL activity *in vivo*. **(A)** Luciferase-transduced P815 cells were injected into lethally irradiated F1 recipients along with TCD-BM or TCD-BM+ allogeneic CD45.1 B6 splenocytes (n=15-18 per group). Recipients of allogeneic splenocytes were treated with vehicle or PLX2853. Survival curve. Data combined from two independent transplant experiments. **(B, C)** Whole-body bioluminescent signal intensity of recipient mice (n= 5 per cohort). Mice were imaged on indicated days. Average radiance expressed as mean ± SD. Data shown is representative of one of two independent transplant experiments. **(D)** Percentage GFP positivity. Splenocytes were isolated at time of death and P815 leukemic burden evaluated by GFP positivity using flow cytometry. **(E)** Splenocytes from vehicle and PLX2853 cohort transplant recipients were analyzed by flow cytometry to evaluate IFNγ secretion and CD107a expression on CTL. **(F)** Representative contour plots. **p < 0.01, ****p < 0.0001. NS, not significant.

It is critical for an acute GVHD therapy to maintain the GVL response since the primary goal of allogeneic HCT is the induction of a donor anti-tumor response to eliminate residual malignant cells in the recipient. Therefore, we investigated the effect of BET inhibition on GVL using a luciferase-transduced GFP+ murine mastocytoma P815 cell line in the B6 into B6D2F1 model. Lethally irradiated B6D2F1 mice received P815 cells along with TCD-BM from B6 mice. Twenty-four hours later, a cohort of mice was injected with allogeneic B6 splenocytes. Recipients of allogeneic splenocytes were then treated with vehicle or PLX2853, starting day +2 post-transplant. Recipients of allogenic splenocytes demonstrated significantly improved survival when compared with mice receiving P815 + TCD-BM alone (**Figure 4A**), regardless of PLX2853 or vehicle treatment. In addition, mice receiving PLX2853 showed reduced luminescence compared to P815+ TCD-BM alone mice with no difference in luminescence when compared with vehicle treated mice (**Figures 4B, C**). Flow cytometric analysis of GFP+ P815 tumor burden in splenocytes of recipient mice confirmed that cause of death in mice receiving allogeneic B6 splenocytes (vehicle and PLX2853 cohorts) was not leukemia/tumor (**Figure 4D**). To evaluate whether PLX2853 impacts cytotoxic T lymphocyte (CTL) function of donor CD8+ T cells, splenocytes from vehicle and PLX2853 treated mice were isolated, and IFN-γ production and degranulation was analyzed. Donor CD45.1+ CD8+ T cells from mice treated with PLX2853 and vehicle showed comparable expression of IFNγ and CD107a suggesting that BET inhibition does not disrupt the CTL function that is critical for GVL effect (**Figures 4E, F**).

### BET Inhibition Significantly Impacts Inflammation Associated Gene Expression Signatures

**Figure 5:**
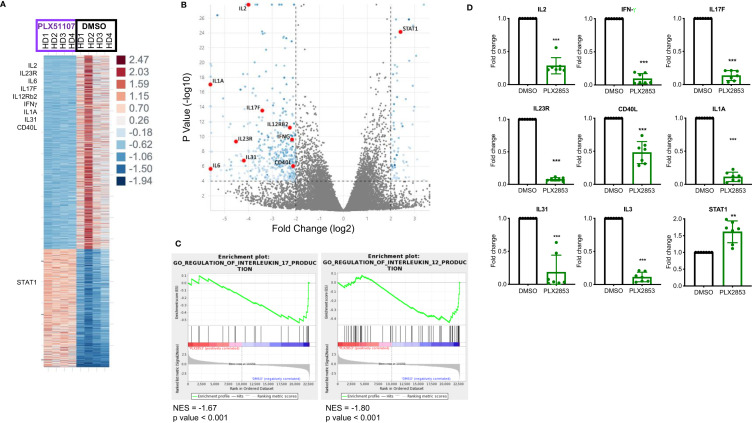
BET inhibition significantly impacts inflammation associated gene expression signatures. Human T cells were isolated from healthy PBMCs (n=4). T cells were stimulated with CD3/CD28 Dynabeads ± PLX51107 (500nM) for 48hrs, RNA isolated and RNA-seq performed. **(A)** Heat map of differentially expressed genes with at least 2-fold change, p<0.05. **(B)** Volcano plot of the top dysregulated genes with at least 4-fold change, p<0.0001. **(C)** GSEA analysis of RNA-seq counts. Gene set enrichment analysis (GSEA) enrichments plots of IL-17 and IL-12 gene expression signatures. **(D)** Validation of RNA-seq data in additional T cells isolated from healthy PBMCs (n=7) by real-time qPCR for indicated genes. T cells were treated with DMSO or PLX2853 (10nM) for 48hrs. Fold change compared to DMSO, gene expression normalized to β-actin. **p < 0.01, ***p < 0.001.

To further characterize the effect of BET inhibition on T cells, we compared gene expression profiles of human T cells stimulated by CD3/CD28 in the presence or absence of PLX51107 using RNA-sequencing. We found multiple inflammatory Th1/Th17-associated genes such as IL-2, IL-23R, IL-6, IL-17F, IFNγ, IL12Rβ2, CD40L downregulated upon treatment with PLX51107 (**Figures 5A, B**). Correspondingly, gene set enrichment analysis (GSEA) highlighted the downregulation of genes associated with the IL-12 and IL-17 signaling pathways (**Figure 5C**), validating earlier studies that showed a role for BET proteins and Th1/Th17 pathways (23, 44–47). Reduced IL-2 expression deviates from a previous BET inhibitor study in which the authors observed an increase in IL-2 expression upon BET inhibition (26). Interestingly, STAT1 expression was significantly higher in the PLX51107 samples compared with DMSO treatment (**Figures 5A, B**). Using T cells treated with the second-generation BET inhibitor PLX2853, we further validated the results by performing quantitative real-time PCR analysis of a smaller subset of genes. Again, we observed significant reductions in gene expression of IL-2, IFN-γ, IL-17F, IL-23R, and CD40L in cells treated with PLX2853, as well as increased expression of STAT1 (**Figure 5D**).

### BET Inhibition Modulates the IL-23/STAT3 Immune Axis and Inhibits T Cell Infiltration Into the GI Tract

**Figure 6:**
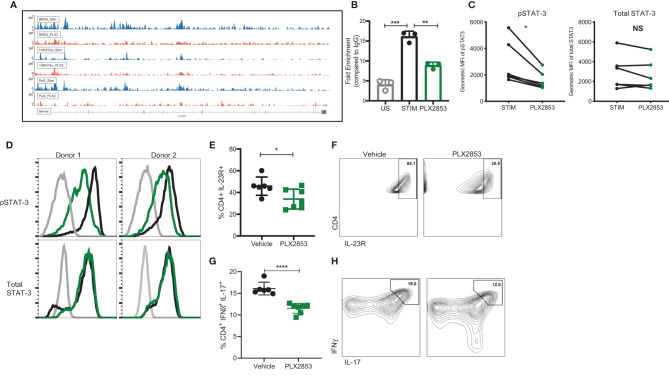
BET inhibition modulates IL-23R/STAT3 axis. **(A)** Healthy human donor (HD) T cells were stimulated with CD3/CD28 for 24 hours in the presence or absence of 10 nM PLX2853. ChIP-sequencing peaks for IL-23R gene. **(B)** ChIP DNA was amplified with real-time qPCR using SYBR Green. Input standard curves were used for estimation of relative enrichment. **(C)** Healthy donor human PBMCs (n=6) were stimulated with CD3/CD28 for 48 hrs ± PLX2853 (10nM, green). Cells were starved for 4 hrs and then pulsed with IL-23 for 15 min, phosphorylated and total STAT3 expression analyzed by flow cytometry. Geometric mean fluorescence intensity (MFI) of phospho- and total STAT3. **(D)** Histogram of two representative donors showing unstimulated (gray), CD3/CD28 stim +IL-23 pulse (black), CD3/CD28 stim + PLX2853 + IL-23 pulse (green). **(E–H)** B6 into B6D2F1 transplant was performed as described earlier and recipients of allogeneic splenocytes were treated with vehicle or PLX2853. Small intestine of the GI tract was harvested from mice as described in methods (n=6 per cohort). **(E)** Percentage and **(F)** contour plots of IL-23R+ CD4+ and **(G)** Percentage and **(H)** contour plots of IFNγ+ IL-17+ CD4+ T cells as determined by flow cytometric analysis. *p < 0.05, **p < 0.01, ***p < 0.001, ****p < 0.0001. Data shown is representative of one of two independent transplant experiments. NS, not significant.

To investigate the molecular mechanisms that gave rise to the transcriptional signatures associated with PLX2853 treated T cells, we performed Chromatin Immunoprecipitation (ChIP)-sequencing on CD3/CD28 stimulated human T cells in the presence or absence of PLX2853. We evaluated BRD4 binding, RNA polymerase II (POL II) occupancy and H3K27Ac (BRD4-associated histone mark) density. We observed a reduction in BRD4 binding to the IL-23R gene, with a corresponding decrease in H3K27ac as well as RNA polymerase II occupancy (**Figure 6A**). Using ChIP-PCR, we validated the reduced binding of BRD4 to the IL-23R gene in PLX2853 treated human T cells (**Figure 6B**). This supports our RNA-seq data in which IL-23R gene expression is downregulated in T cells upon treatment with either pharmacological inhibitors PLX51107/PLX2853 and small interfering RNA (siRNA) knockdown of BRD4 (**Figure S4**). In order to determine whether the reduction in IL-23R expression has functional consequences in downstream signaling events, we evaluated phosphorylation of STAT3 (p-STAT3) in response to exogenous IL-23 stimulation in human T cells in the presence of PLX2853. Flow cytometric analysis revealed a significant decrease of STAT3 phosphorylation in the presence of BET inhibitors PLX51107 (**Figure S5**) or PLX2853 without significant changes in total STAT3 expression (**Figures 6C, D**). Complementary to our *in vitro* findings in human T cells, we show that PLX2853 administration *in vivo* resulted in significant downregulation of IL-23R expression (**Figures S6A, B**) as well as reduced STAT3 phosphorylation (but not total STAT3) in response to IL-23 stimulation (**Figures S6C, D**). IL-23 is known to drive intestinal T cell proliferation and promote the accumulation of Th17 cells in the intestines (48). Therefore, we sought to determine if BET inhibition modulated IL-23R expression and Th17 infiltration in the GI tract of allogeneically transplanted B6D2F1 recipients *in vivo*. We observed a significant reduction in the percentage of IL-23R+ CD4+ T cells in colonic intraepithelial lymphocytes (IELs) of PLX2853-treated mice in comparison to vehicle (**Figures 6E, F**). In addition, an IFNγ+ IL-17+ double positive CD4+ T cell population known to arise in response to IL-23R signaling (48) was also decreased in the colonic IELs of mice treated with PLX2853 (**Figures 6G, H**). Altogether, these data support our assertion that BRD4 regulates Th17 pathogenicity through IL-23R and the IL-23/STAT3 pathway.

## Discussion

Herein, we demonstrate that BET inhibition with PLX51107/2853 dampens the T cell inflammatory response following allogeneic BMT *via* modulation of IL-23R/STAT3 pathway, significantly improving survival in animal models without evidence of impaired GVL or Treg function. We show that BET inhibition with PLX51107/2853 decreases the binding of BRD4 as well as H3K27ac and RNA Pol II occupancy to the IL-23R gene in human T cells with concomitant decrease in IL-23R expression and signaling in both human T cells *in vitro* and murine T cells *in vivo*. These findings suggest that there exists a direct interaction between BRD4 and the IL-23R locus which is disrupted by PLX51107/2853, resulting in the decreased inflammatory response. Our studies confirm previous findings wherein allogeneic donor T cell proliferation, inflammatory cytokine secretion and DC maturation (23, 26, 27, 49, 50) are suppressed upon BET inhibition, thereby solidifying a therapeutic role for BET inhibitors such as PLX51107 and PLX2853 in acute GVHD.

In recent years, the Th17 subset has been identified as critical in the pathogenesis of acute GVHD along with the Th1 population (10). Additionally, Th17 pathogenicity has been linked to STAT3 signaling through IL23R, prompting investigation into the role of IL-23R in acute GVHD (12, 51, 52). Suppression of Th17 differentiation *via* inhibition of STAT3 pathway has been shown to reduce IL-17 and RORγt expression in murine experimental autoimmune myocarditis while simultaneously increasing the expression of FOXP3 (53). Previous work has shown that IL-23R signaling is required by Th17 cells in order to become pathogenic (54, 55); however, the effect of BET proteins on the IL-17/IL-23 immune axis especially in the context of acute GVHD remained unexplored. The GI tract is a key target organ of acute GVHD, and tissue damage mediated by T cells in this organ is associated with acute GVHD-related mortality (2, 15, 56). Direct signaling through the IL-23R in T cells has been shown to drive their proliferation to promote accumulation of intestinal Th17 cells (48) as well as other Th1 pathogenic populations (57). Our studies have revealed a direct role for BET proteins regulating the pathogenic IL-23R/STAT3/IL-17 axis. Our *in vitro* and *in vivo* experiments have demonstrated that BET inhibition using PLX51107/2853 downregulates IL-23R expression and function as evidenced by decreased phosphorylation of STAT3 in response to IL-23 stimulation in both human (*in vitro*) and mouse T cells (*in vivo*). We also observed a reduction of a distinct population of CD4+ IFN-γ+ IL-17+ T cells in colonic IELs upon BET inhibition, a pathogenic population shown to emerge in response to IL-23 signaling (48). Importantly, our ChIP-seq analysis shows a clear reduction of BRD4 binding to the IL-23R gene upon treatment with PLX2853, illustrating the direct effect of BRD4 on IL-23R. To our knowledge, this is the first time it has been shown that BRD4 directly acts upon the IL-23R gene to modulate the pathogenic Th17 response.

Our observations of reduced production of pro-inflammatory cytokines IFN-γ and TNF-α as well as IL-17 by donor T cells indicate that PLX51107 and PLX2853 are effectively targeting both the Th1 and Th17 subsets. These effects are likely responsible for the observed acute GVHD clinical response and improved survival. It will be interesting to explore whether the reduction in Th1 and Th17 phenotypes corresponds to an emergence of a larger Th2 population.

While acute GVHD is mediated by T cells, the role of APCs in the pathogenesis of the disease cannot be ignored. Induction of acute GVHD occurs when APCs from the recipient encounter donor T cells, which leads to the expansion, differentiation, and migration of these T cells (1, 3, 4, 6). Our results have shown that PLX51107 and PLX2853 have profound effects on dendritic cell maturation and cytokine secretion *in vitro* and *in vivo.* These findings combined with the effects on T cell inflammatory response show that these compounds have the ability to combat GVHD pathogenesis at both the effector and responder levels. Further investigation is required to elucidate the mechanism behind the observed effects on APCs. Importantly, we also show that BET inhibition *via* PLX51107/2853 preserves Tregs, a population responsible for dampening inflammatory activity of other Th subsets.

An intriguing finding from this study was upregulation of STAT1 upon BET inhibition with PLX51107 and PLX2853, which is in line with previous studies (58, 59). This asserts that the anti-inflammatory effects of BRD4 inhibition are independent of STAT1, which is a target of the currently FDA approved Jak1/2 inhibitor, ruxolitinib, for steroid refractory acute GVHD. Thus, potential benefits may be conferred by combination therapy with BRD4/JAK inhibition. This strategy is currently being employed in treatment of myeloproliferative disorders (60, 61), and it will be interesting to uncover the effects of this treatment in acute GVHD. Our group is currently investigating the effects of combined BRD4 and JAK dual inhibition in mouse models of aGVHD.

In conclusion, we describe the potent anti-inflammatory effects of BET inhibition using PLX51107/2853 in mouse models of aGVHD and GVL. Given these promising preclinical results, PLX51107 will enter clinical trial for refractory acute GVHD in a Phase 1 safety, biological efficacy analysis at our center (NCT04910152). Furthermore, studies from the correlative analyses will examine the clinical efficacy of IL-23/STAT3 inhibition with this novel BET inhibitor.

## Data Availability Statement

The original contributions presented in the study are publicly available in NCBI using accession number PRJNA757357 and the GEO database with accession GSE183883 and GSE183884.

## Ethics Statement

The animal study was reviewed and approved by Institutional Animal Care and Use Committee Ohio State University.

## Author Contributions

KS, YG, NS, NZ, and LN-C performed in-vivo murine acute GVHD experiments, ex-vivo FACS analyses, serum/supernatant ELISAs, real-time PCR analyses, *in-vitro* experiments with mouse cells and human PBMCs, RNA-seq and ChIP-seq, signaling experiments, analyzed the data and interpreted the results. KS and KB performed the BRD4 siRNA experiments. SK and AD performed histopathological analysis. AD and MM performed IHC staining and ISH and analyzed the results. SS and LS performed ChIP-PCR experiments and analyzed the results. HC analyzed data, discussed experimental design, and edited the manuscript. KS, HC, and PR wrote the manuscript. SD, GB, YM, and BP provided discussion and edited the manuscript. PR designed the study, supervised research, analyzed and interpreted the data, and edited the manuscript. All authors contributed to the article and approved the submitted version.

## Funding

This work was supported by Division of Hematology and OSUMC start-up funds (PR and HC), Division Sponsored Research Program (PR and HC), CCTS Core Services Voucher (PR), K12 Paul Calabresi Award (PR) and NCI R01CA252469-01A1 (HC and PR). MM is supported by NIH R00 AG054760, the American Federation of Aging Research (AFAR) and OSUMC startup funds. Research reported in this publication was supported by the Ohio State University Comprehensive Cancer Center and the National Institutes of Health under grant number P30 CA016058. We thank the Flow Cytometry Shared Resource at The Ohio State University Comprehensive Cancer Center, Columbus, OH for analytical cytometry. This study received funding from Plexxikon Inc. The funder was not involved in the study design, collection, analysis, interpretation of data, the writing of this article or the decision to submit it for publication. All authors declare no other competing interests.

## Conflict of Interest

GB, YM, and BP are employees of Plexxikon Inc.

The remaining authors declare that the research was conducted in the absence of any commercial or financial relationships that could be construed as a potential conflict of interest.

The handling editor declared a shared affiliation with the authors at time of review.

## Publisher’s Note

All claims expressed in this article are solely those of the authors and do not necessarily represent those of their affiliated organizations, or those of the publisher, the editors and the reviewers. Any product that may be evaluated in this article, or claim that may be made by its manufacturer, is not guaranteed or endorsed by the publisher.
