# Supplementary material for: Inhibition of Bromodomain and Extra Terminal (BET) Domain Activity Modulates the IL-23R/IL-17 Axis and Suppresses Acute Graft-Versus-Host Disease
Source: Front Oncol. 2021 Oct 15;11:760789. doi: 10.3389/fonc.2021.760789 (PMC8554203; doi:10.3389/fonc.2021.760789)
Supplement: Supplementary file 1 [file DataSheet_1.pdf]

Data supplement for Inhibition of Bromodomain and Extra Terminal (BET) domain activity modulates the IL-23R/IL17 axis and is a therapeutic target for acute Graft-Versus-Host Disease

1. Supplemental Methods.
2. Supplemental Figures – Figures S1 – S7.
3. Supplemental Table – Table S1.

1. Supplemental Methods

Cells and cell culture

Mouse B6 or CD45.1 B6 T cells were isolated from splenocytes using Pan-T Cell Isolation Kit or Naïve CD8 T cell isolation kit (Miltenyi Biotec) per manufacturer's protocol. All cells were cultured in RPMI 1640, 20% FBS, and 1% Pen-Strep unless otherwise specified. Healthy human donor buffy coats were procured from Versiti and PBMCs isolated by Ficoll-Paque PLUS density gradient centrifugation (GE Healthcare). T cells were isolated from PBMCs using Pan-T Cell Isolation Kit (Miltenyi Biotec).

In vitro T cell proliferation:

CD45.1 B6 T cells and human T cells were labeled with Cell Trace Violet (CTV) and incubated with allogeneic BALB/c bone marrow derived dendritic cells (BMDCs) or stimulated using CD3/CD28 DynaBeads (Life Technologies). Cell division was measured by CTV dilution after 4-5 days using LSRII and FACS Diva software (Becton Dickinson).

Dendritic cell assays

Bone marrow cells were harvested from the femurs and tibiae of BALB/c mice. Erythrocytes were lysed using ammonium chloride, and cells were cultured in RPMI containing 10% FBS, 1% penicillin-streptomycin, granulocyte-macrophage colony-stimulating factor (GM-CSF; PeproTech, 20 ng/mL), and IL-4 (PeproTech, 10 ng/mL). After three days in culture, half of the media was replaced including fresh cytokines. After five total days in culture, immature BMDCs were washed and resuspended in RPMI containing 10% FBS and 1% penicillin-streptomycin and incubated with 5 ug/mL lipopolysaccharide (LPS) for 6 hours to induce maturation in the presence of either DMSO, PLX51107, or PLX2853 then harvested for flow cytometric analysis of maturation markers. Supernatants were collected for cytokine ELISA.

Cytokine ELISA

Mouse and human T cells as well as murine BMDCs were stimulated as described above. Supernatant cytokines were analyzed by ELISA according to manufacturer's protocol (BioLegend).

Small interfering RNA (siRNA) transfection

T cells isolated from healthy donor human PBMCs were stimulated with CD3/CD28 DynaBeads for 72 hours. At 72 hours, DynaBeads were removed, and cells were transfected with siRNAs specific to BRD4 or a scramble non-targeting control using a Neon Transfection System (Invitrogen). All siRNAs were used at a concentration of 200 nM. For the period of incubation following transfection, CD3/CD28 DynaBeads were added to cells to maintain viability. RT qPCR was performed 72 hours after transfection to confirm effects of BRD4 siRNA knockdown.

Figure S1

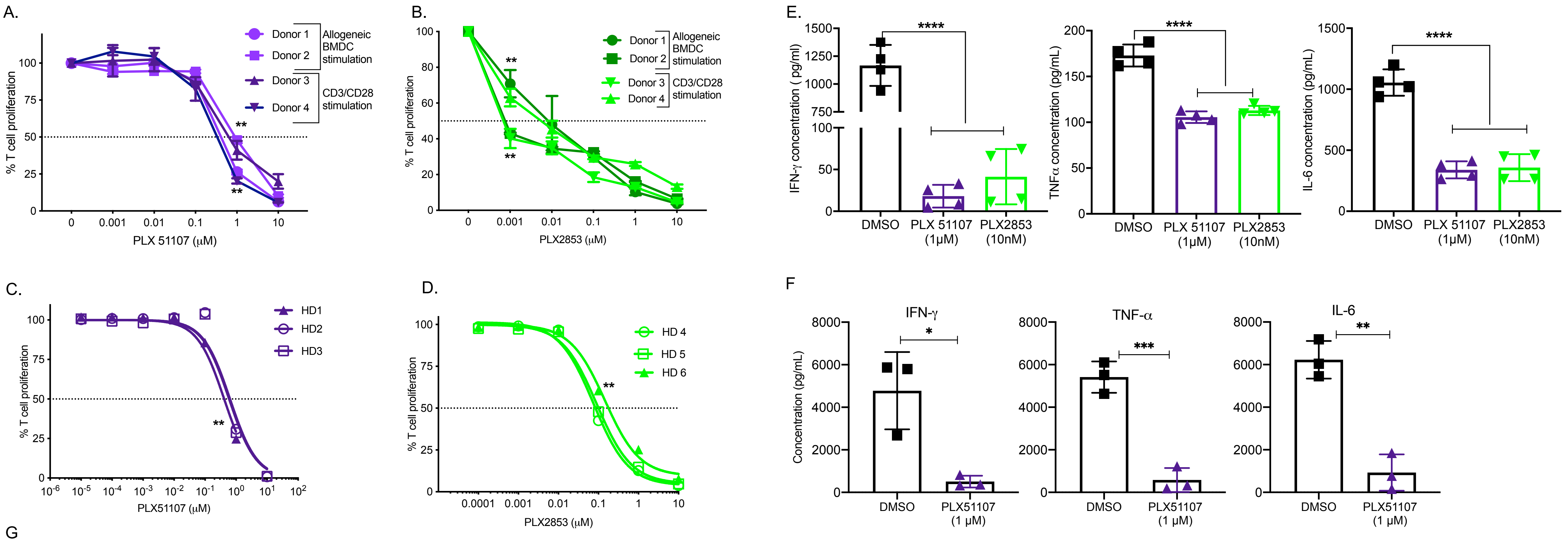

G

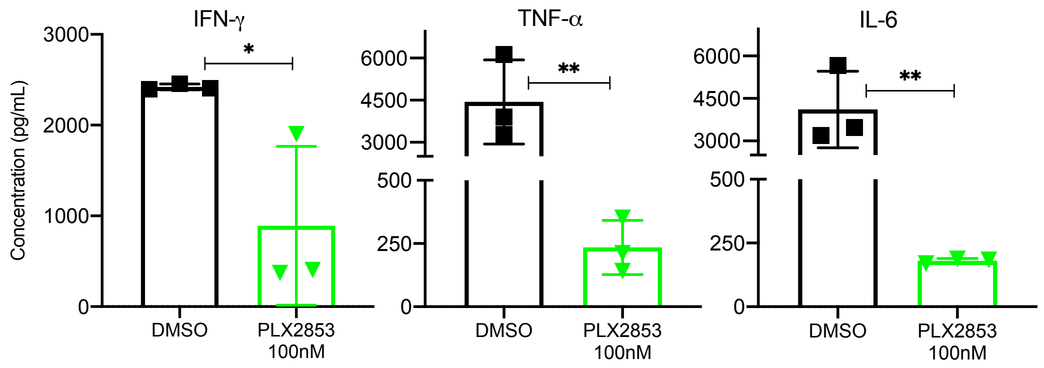

Supplemental Figure 1. BET inhibitors PLX51107 and PLX2853 significantly reduce mouse and human T cell inflammatory response. Cell trace violet (CTV) labeled B6 T cells stimulated with allogeneic BALB/c BMDCs or CD3/CD28 beads for 4 days ± **(A)** PLX51107 or **(B)** PLX2853. T cell proliferation analyzed by CTV dilution via flow cytometry, normalized to DMSO. CTV labeled healthy donor human T cells stimulated with CD3/CD28 beads for 4 days ± **(C)** PLX51107 or **(D)** PLX2853. **(E)** Murine supernatants assayed for indicated cytokines using ELISA. Human supernatant ELISA for **(F)** PLX51107 and **(G)** PLX2853. For CTV proliferation assays and cytokine ELISA, n=3-4 donors. In all experiments, * $p<0.05$, ** $p<0.01$, *** $p<0.001$, **** $p<0.0001$ compared to DMSO control.

Figure S2

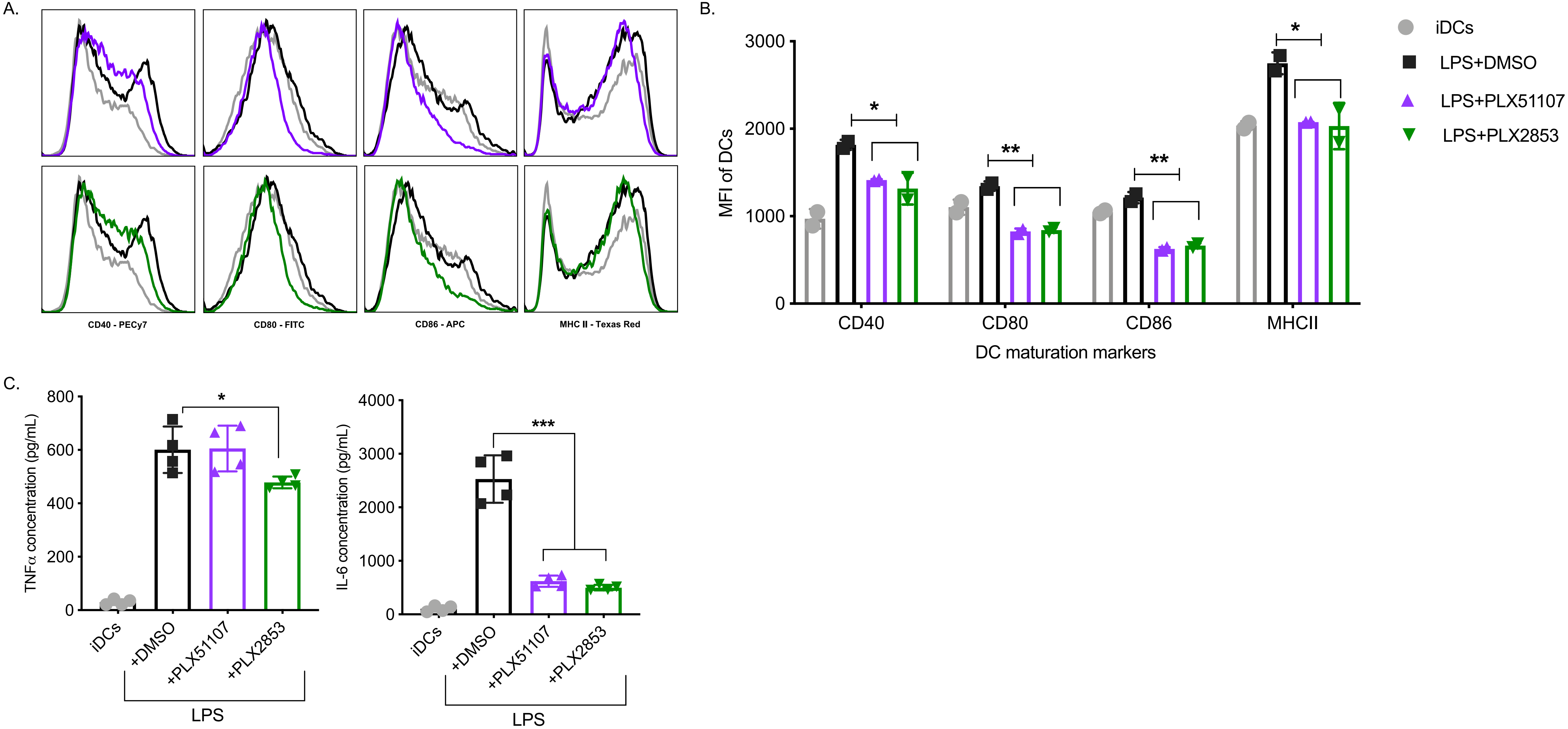

Supplemental Figure 2. BET inhibitors (PLX) significantly reduce DC maturation and activation. Immature murine BMDCs (iDCs) were stimulated with LPS (5ug/ml, 6 hours) to induce maturation ± PLX51107 (500nM) or PLX2853 (10nM). Flow cytometric evaluation of maturation markers – CD40, CD80, CD86 and MHC II on CD11c+ DCs. **(A)** Representative histograms. **(B)** Mean fluorescence intensity of maturation markers, MFI. **(C)** Supernatants assayed for cytokines using ELISA. Data is combined from two independent experiments performed in duplicate. * $p<0.05$, ** $p<0.01$ compared to LPS+DMSO treatment.

Figure S3

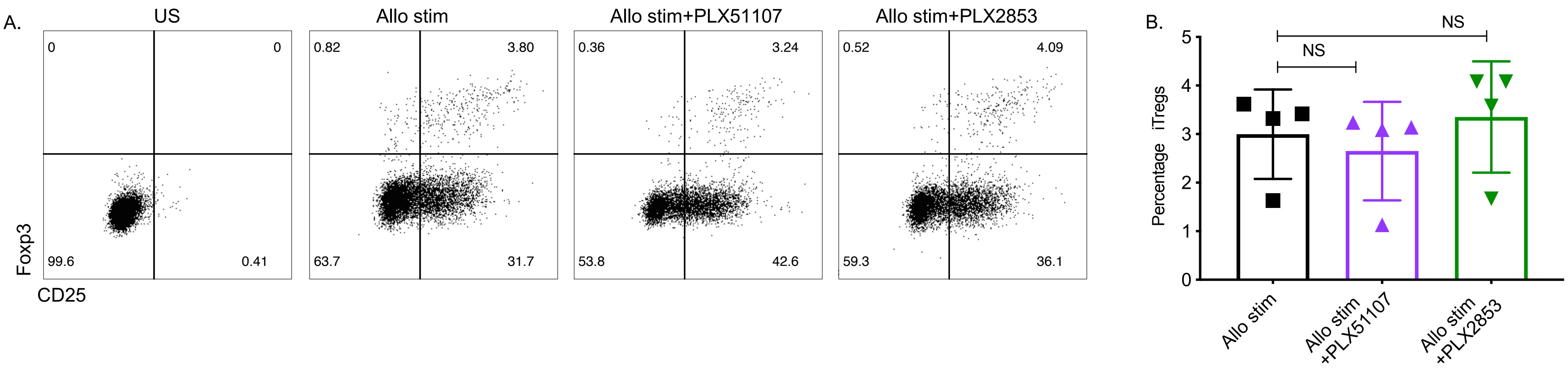

Supplemental Figure 3. BET inhibition does not sacrifice Tregs. BoyJ CD4+ CD25- cells were co-cultured with Balb/c BMDCs (4:1) ± PLX51107 (250nm) or PLX2853 (10nM) for 5 days. Inducible Treg (iTreg) cells (CD25+ Foxp3+) were measured by flow cytometry. **(A)** Representative contour plots of CD25 versus Foxp3. **(B)** Graph shows frequency of inducible Treg cells after 5 days of DC-allostimulation with or without PLX. Mean ± S.D., data is combined from four independent experiments.

Figure S4

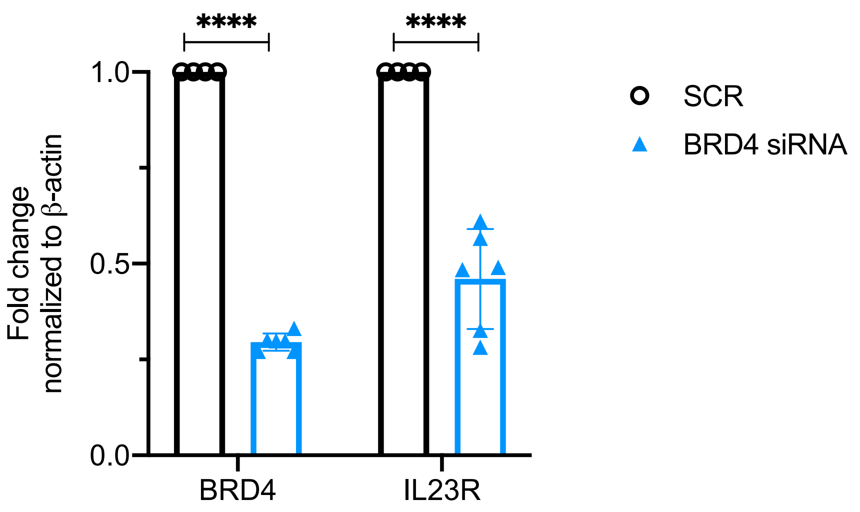

Supplemental Figure 4. Genetic knock-down of BRD4 downregulates IL-23R expression. T cells isolated from healthy donor human PBMCs were stimulated with CD3/CD28 DynaBeads and transfected with SCR control or BRD4 siRNA. Cells were harvested 72 hours later, RNA isolated and real-time qPCR analysis was performed for the indicated genes. Mean ± S.D., data is pooled from 3 independent experiments.

Figure S5

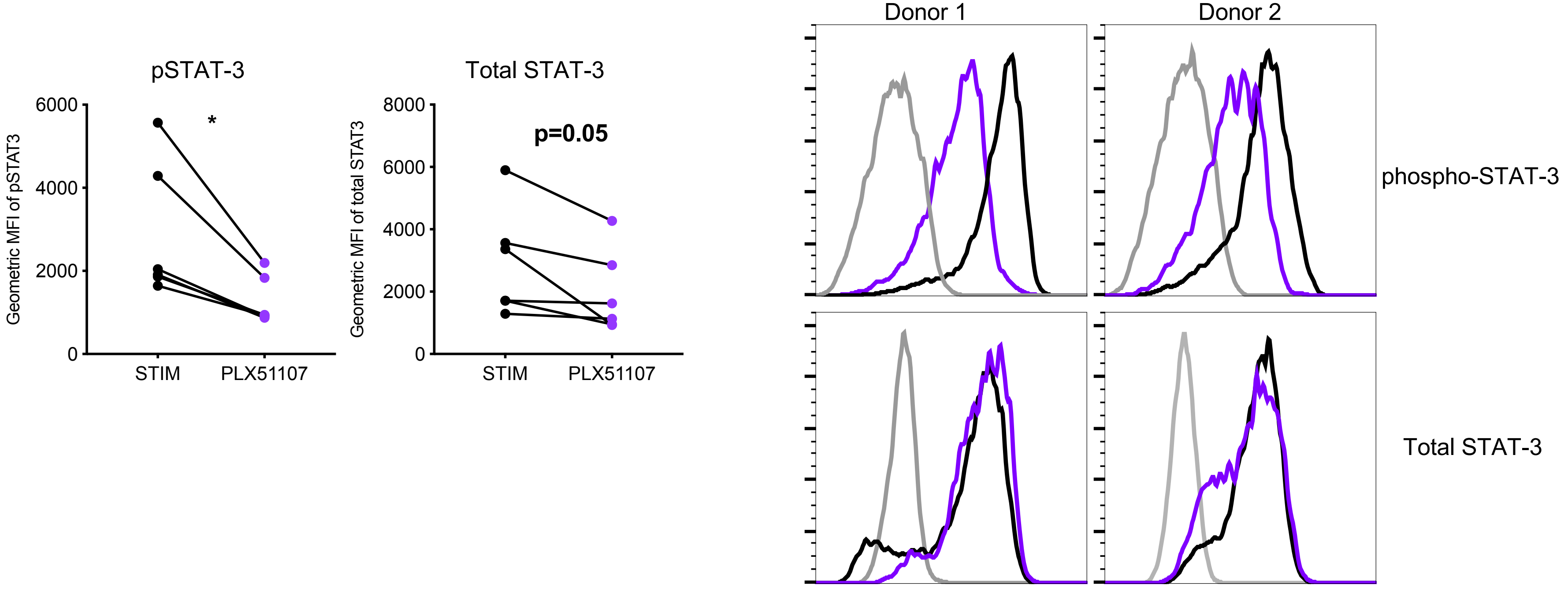

Supplemental Figure 5. PLX51107 downregulates IL23 mediated STAT3 phosphorylation in human T cells. Healthy donor human PBMCs (n=6) were stimulated with CD3/CD28 for 48 hrs ± PLX51107 (250nM, purple). Cells were starved for 4 hrs and then pulsed with IL-23 for 15 min, phosphorylated and total STAT3 expression analyzed by flow cytometry. **(A)** Geometric mean fluorescence intensity (MFI) of phospho- and total STAT3. **(B)** Histogram of two representative donors showing unstimulated (gray), CD3/CD28 stim +IL-23 pulse (black), CD3/CD28 stim + PLX51107 + IL-23 pulse (purple). * $p<0.05$.

Figure S6

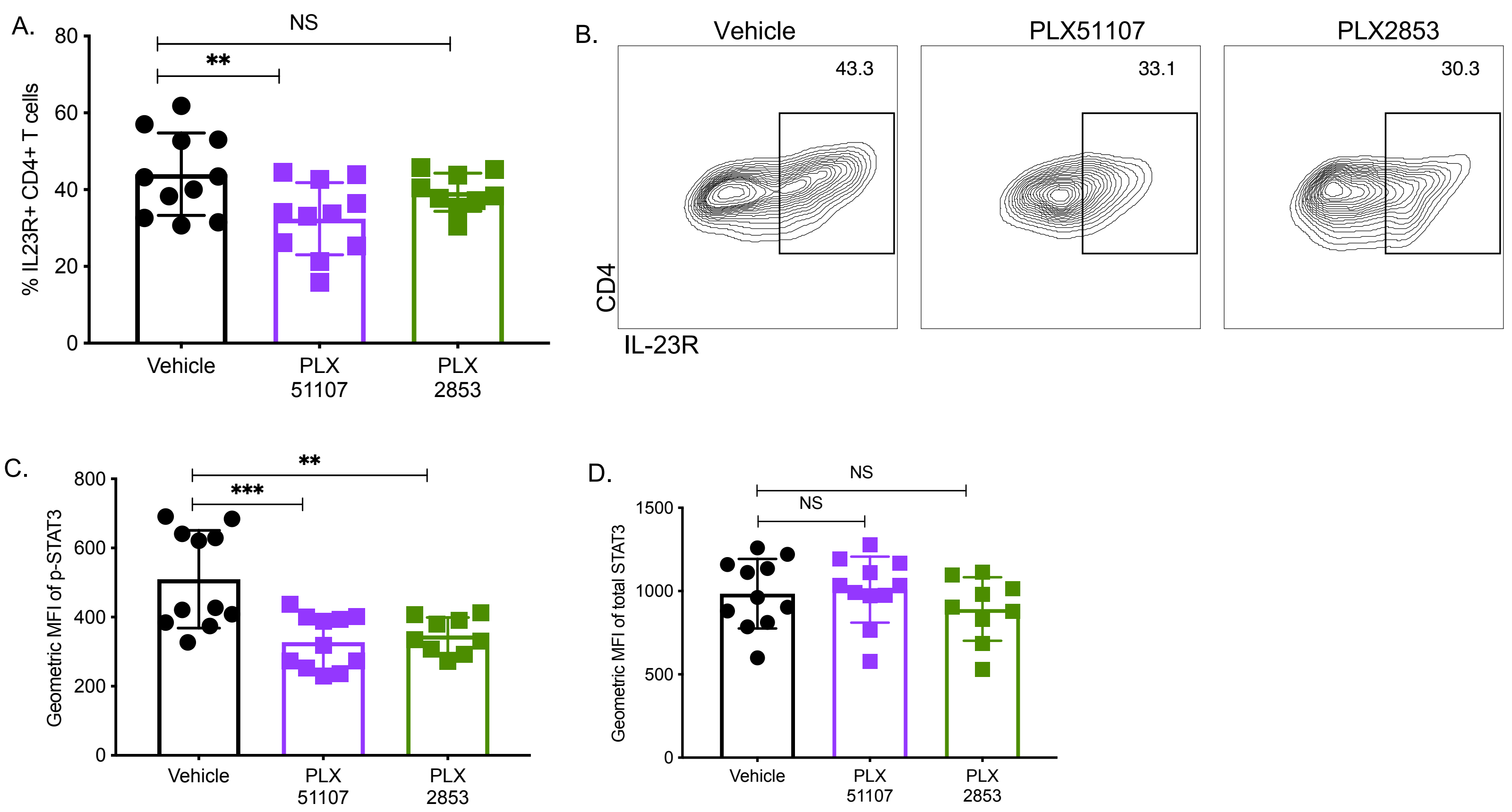

Supplemental Figure 6. BET inhibition modulates IL-23R expression and IL-23 mediated STAT3 phosphorylation in vivo. CD45.1 B6 into B6D2F1 transplant was performed as described in methods and recipients of allogeneic splenocytes were treated with vehicle, PLX511107 or PLX2853 (n= 9-11 per cohort). Mice were euthanized between days 25-28 post-transplant, splenocytes harvested. **(A)** Percentage IL23R+ CD4+ T cells analyzed by flow cytometry. **(B)** Representative contour plots. Splenocytes were stimulated with IL-23 (50ng/ml) for 40 min at 37°C. Mean fluorescence intensity (MFI) of **(C)** phospho-STAT3 and **(D)** total STAT3 in CD4+ T cells. Data pooled from three independent transplants. Each symbol represents an individual mouse.

Figure S7

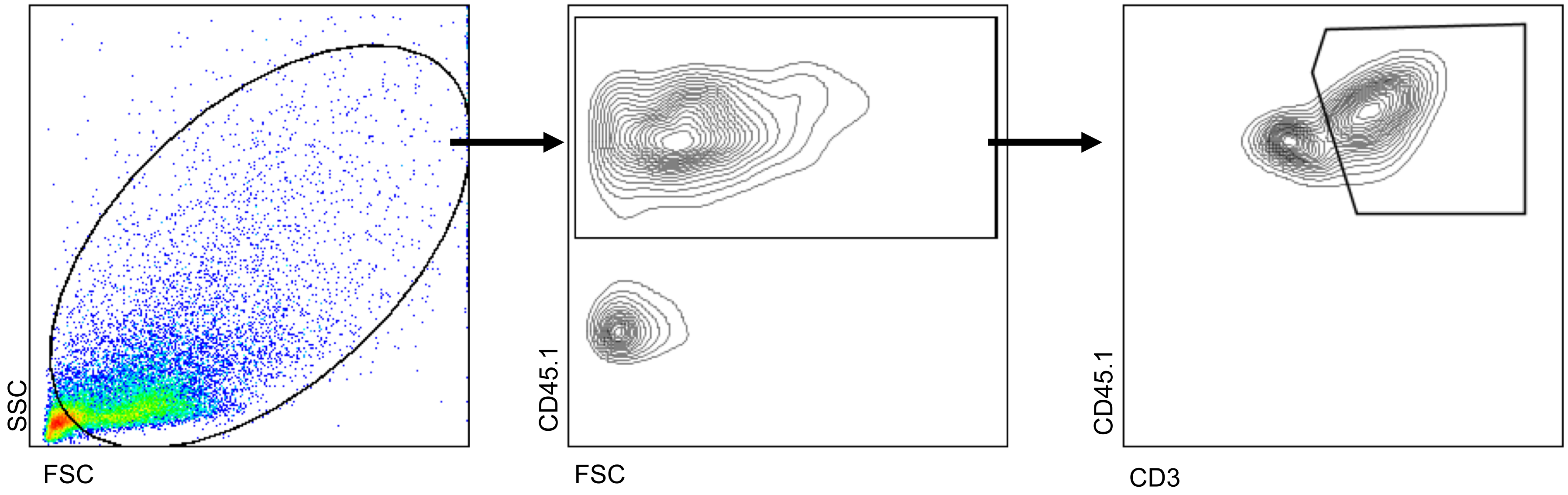

Supplemental Figure 7. Gating Strategy for Flow Cytometry Experiments. Lymphocyte population is gated on in SSC x FSC plots. From this population CD45.1+ donor cells are selected. From within CD45.1+ donor cells, CD3+ T cells are selected.

Supplemental Table S1. List of antibodies used

Target	Clone	Fluorochrome	Vendor
CD3 (m)	145-2C11	FITC	BioLegend
CD3 (m)	17A2	APC/Cyanine7	BioLegend
CD3 (h)	HIT3a	FITC	BioLegend
CD3 (h)	OKT3	Brilliant Violet 421	BioLegend
CD4 (h)	A161A1	FITC	BioLegend
CD4 (m)	GK1.5	Brilliant Violet 605	BioLegend
CD4 (h)	OKT4	Pacific Blue	BioLegend
CD4 (m)	RM4-5	PE/Cyanine5	BioLegend
CD8 (h)	SK1	PE/Cyanine7	BioLegend
CD8a (m)	53-6.7	Brilliant Violet 421	BioLegend
CD11c (m)	N418	Brilliant Violet 421	BioLegend
CD25 (m)	PC61	PE/Cyanine7	BioLegend
CD40 (m)	1C10	PerCP-eFluor710	Invitrogen – Thermo Fisher
CD40 (m)	FGK45.5	PE-Vio770	Miltenyi Biotec
CD45.1 (m)	A20	Brilliant Violet 421, PE	BioLegend
CD80 (m)	16-10A1	FITC	BioLegend
CD86 (m)	GL-1	APC	BioLegend
CD107a (m)	REA777	APC	Miltenyi Biotec
FOXP3 (m)	FJK-16s	PerCP/Cyanine5.5	Invitrogen – Thermo Fisher
FOXP3 (m)	MF-14	Alexa Fluor 647	BioLegend
IFN γ(m)	XMG1.2	FITC	BioLegend
IL-17A (m)	TC11-18H10.1	Brilliant Violet 605	BioLegend
IL-23R (m)	12B2B64	APC	BioLegend

Ki67 (m)	SolA15	PE/Cyanine7	Invitrogen – Thermo Fisher
MHC II (m)	M5/114.15.2	APC	eBioscience – Thermo Fisher
MHC II (m)	REA813	PE-Vio615	Miltenyi Biotec
Phospho-STAT3 (h, m)	13A3-1	Alexa Fluor 647	BioLegend
TNFα (m)	MP6-XT22	APC	BioLegend
TNFα (m)	MP6-XT22	FITC, PerCP-eFluor710	Invitrogen – Thermo Fisher
Total STAT3 (h)	15H2B45	PE	BioLegend
